# Utilization of Acidic *α*-Amino Acids as Acyl Donors: An Effective Stereo-Controllable Synthesis of Aryl-Keto *α*-Amino Acids and Their Derivatives

**DOI:** 10.3390/molecules19056349

**Published:** 2014-05-16

**Authors:** Lei Wang, Yuta Murai, Takuma Yoshida, Masashi Okamoto, Zetryana Puteri Tachrim, Yasuyuki Hashidoko, Makoto Hashimoto

**Affiliations:** 1 Division of Applied Bioscience, Graduate School of Agriculture, Hokkaido University; Kita 9, Nishi 9, Kita-ku, Sapporo 060-8589, Japan; 2 Faculty of Advanced Life Science, Frontier Research Center for Post-Genome Science and Technology, Hokkaido University, Kita 21, Nishi 11, Kita-ku, Sapporo 001-0021, Japan

**Keywords:** aryl-keto *α*-amino acid, Friedel-Crafts acylation, Lewis acid, triflic acid

## Abstract

Aryl-keto-containing *α*-amino acids are of great importance in organic chemistry and biochemistry. They are valuable intermediates for the construction of hydroxyl *α*-amino acids, nonproteinogenic *α*-amino acids, as well as other biofunctional components. Friedel-Crafts acylation is an effective method to prepare aryl-keto derivatives. In this review, we summarize the preparation of aryl-keto containing *α*-amino acids by Friedel-Crafts acylation using acidic *α*-amino acids as acyl-donors and Lewis acids or Brönsted acids as catalysts.

## 1. Introduction

**Scheme 1:**
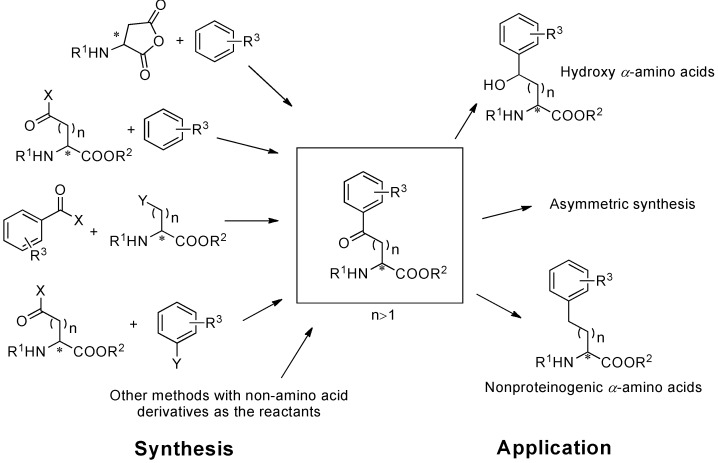
Synthesis and application of aryl-keto *α*-amino acids.

Aryl-keto functional groups are of great importance in organic and biological chemistry. *α*-Amino acids containing aryl-keto functional groups have emerged as versatile and important building blocks for the synthesis of biological compounds, pharmaceutical intermediates, and natural products for several decades [1,2,3,4,5] (Scheme 1). By utilizing optically pure *α*-amino acids, which act as multifunctional and commercially available chiral pools, it is possible to prepare optically pure compounds.

Recently, the synthesis of aryl-keto *α*-amino acids using methods that are straightforward and convenient has attracted more attention. To this end, numerous methods have been developed, such as nucleophilic substitution [6,7], catalytic alkylations [8,9], and Michael additions [10] and so on. Generally, these methods require the preparation of special reagents and expensive catalysts while rigorous conditions are also necessary. Furthermore, the chiral resolution of the enantiomers creates difficulties in the application of these strategies. Friedel-Crafts acylation [11], a method used to acylate aromatic rings, has been widely used in organic synthesis. The corresponding products are less reactive than the original compounds due to the electron-withdrawing effects of the carbonyl group, which hinder multiple acylations. Due to the resonance effect, no carbocation rearrangements occur during the reaction. Construction of aryl-keto *α*-amino acids by Friedel-Crafts acylation has been carried out for many years [12,13,14]. Through the reaction of aromatics and a side chain of *α*-amino acids derivatives, it is easy to synthesize optically pure *α*-amino acids containing enantiomer skeletons. Hydroxy *α*-amino acids and non-proteinogenic *α*-amino acids can be easily prepared, followed by further hydrogenolysis and deprotection. In addition, the enantiomer configuration can be well controlled by using chiral *α*-amino acids as acyl-donors, which is useful for chiral and asymmetric synthesis. It is well known that catalysts such as Lewis acids and Brönsted acids are essential for Friedel-Crafts acylation. The preparation of aryl-keto *α*-amino acids is mainly carried out by the catalysis of AlCl_3_, hydrofluoric acid (HF), or triflic acid (TfOH). This review focuses on the strategies used for the construction of aryl-keto *α*-amino acids by Friedel-Crafts acylation using different catalysts, aiming to provide a comprehensive discussion about alternative methods for the preparation of aryl-keto *α*-amino acids as well as their derivatives.

## 2. General Methods to Prepare Aryl-keto α-Amino Acid Derivatives

In this article, we briefly discuss the general methods used to prepare aryl-keto *α*-amino acid derivatives. The strategies are categorized based on the use of *α*-amino acid derivatives in the reactions.

### 2.1. α-Amino Acid Derivatives as the Reactants

**Scheme 2:**
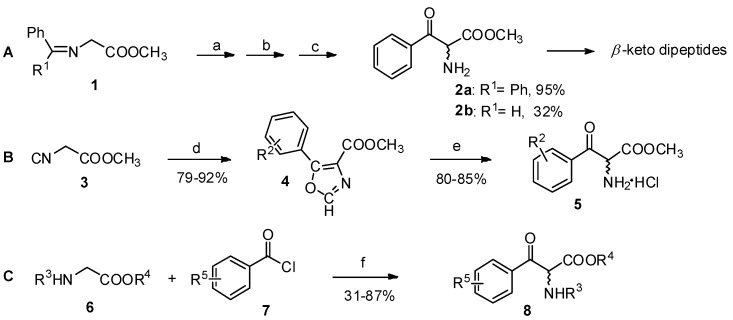
Preparation of aryl-keto *α*-amino acid derivatives by Claisen condensation.

The Claisen condensation is an effective method for the preparation of *β*-keto esters [15]. Reactions of *α*-amino acid derivatives such as glycine Schiff base esters (**1**, Scheme 2A), isocyano esters (**2**, Scheme 2B), or glycine derivatives (**3**, Scheme 2C) with benzyl chloride or aryl anhydride analogs in the presence of bases are procedures for the construction for aryl-keto *α*-amino acid derivatives [16,17,18].

*Reagents and conditions*: (a) KO*_t_-*Bu, THF, −78 °C; (b) PhCOCl; (c) aq. HCl; (d) R^2^COCl or (R^2^CO)_2_O, 1,8-diazabicyclo-[5.4.0]undec-7-ene; (e) MeOH-HCl; (f) (i) *n*-BuLi, THF, −78 °C; (ii) ZnCl_2_, lithium diisopropylamide, −78 °C. R^1^ = Ph, H; R^2^ = 3,4-methylenedioxy-, 3,4,5-trimethoxy-, 3,4-dichloro-; R^3^ = Ts, TFA, Boc; R^4^ = *t-*Bu, Me, Bn; R^5^ = H-, 4-phenyl-, cyclohexyl-.

**Scheme 3:**
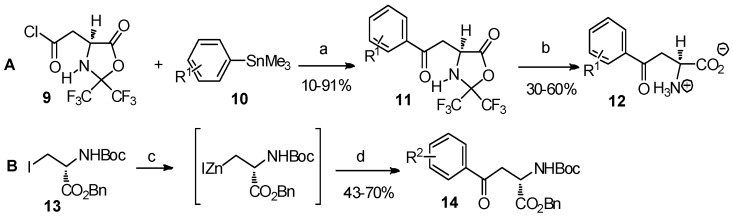
Cross-coupling reaction: a useful method to construct aryl-keto *α*-amino acids.

The cross-coupling reaction is widely used in organic synthesis to directly form a C-C bond between two components. Through the reaction of acyl or alkyl halides of *α*-amino acid derivatives with organic metal compounds, in the presence of palladium catalysts, numerous synthesis of aryl-keto *α*-amino acid derivatives have been reported (Scheme 3) [19,20]. 

*Reagents and conditions*: (a) palladium catalysts: [(Pd(0): Pd_2_(DBA)_3_·CHCl_3_, Pd(PPh_3_)_4_; Pd(II): PhCH_2_Pd(PPh_3_)_2_Cl and (Ph_3_P)_2_PdCl_2_]. (b) H_2_O/*i*-PrOH; (c) Zn/Cu, dimethylacetamide, benzene; (d) R^2^COCl, (Ph_3_P)_2_PdCl_2_. R^1^ = H-, 4-fluoro-; R^2^ = H-, 4-methoxy-, 4-acetoxy-.

**Scheme 4:**
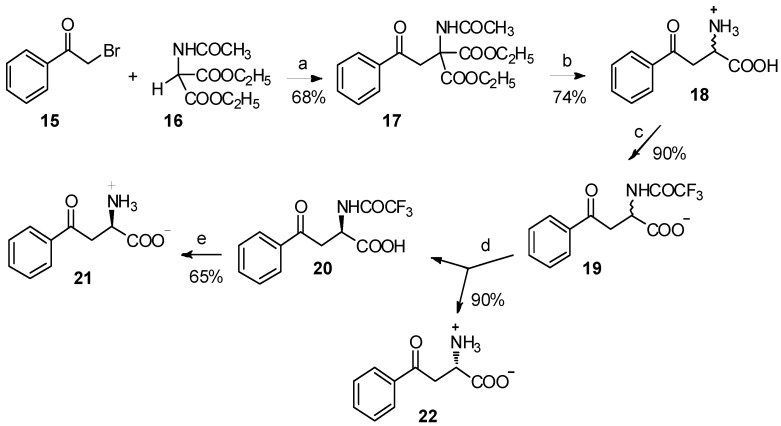
Synthesis of *α*-benzoyl-L-alanine and *α*-benzoyl-D-alanine from acetamidomalonate.

The major barriers to their application are the difficulties in the preparation of organic metal compounds. Acetamidomalonate **16** has been used for the classical synthesis of *α*-amino acids by *C*-alkylation, under basic conditions for many decades [21,22,23,24]. Through the reaction between acetamidomalonate **16** and *α*-bromoacetophenone (**15**), 4-phenyl-4-oxo-2-acetamido-2-ethoxycarbonylbutyrate (**17**) was easily obtained (Scheme 4) [25]. Following acid hydrolysis and enzymatic resolution using carboxypeptidase A digestion, *α*-benzoyl-L-alanine and *α*-benzoyl-D-alanine were isolated in high yield and enantiomeric purity. A more comprehensive study has been reported by Varasi and coworkers [26]. Through the reaction of acetamidomalonate and *α*-chlorocetophenone, a series of kynurenic acid derivatives were prepared. Meanwhile, in the presence of *tert*-butyl lithium, acetamidomalonate could readily react with benzoyl chloride to form the corresponding *β*-keto amino acid derivative [27].

*Reagents and conditions*: (a) NaH, DMF, 0 °C; (b) 6 N HCl, 1,4-dioxane, reflux; (c) ethyl trifluoroacetate, tetramethylguanidine, DMF; (d) carboxypeptidase A, pH = 7.8; (e) NaOH.

In addition, the Grignard reaction of azlactones [28], nucleophilic substitution with phenyl lithium [29,30] or *α*-bromoalanine derivative [31], oxidation of tryptophan [32], reaction of *N*-acylimino acetates and trimethylsilyl enol ethers [33], acylation reaction with lithio dianion [34], reaction of Schiff base esters and silyl enol ether of acetophenone [35] are also useful methods for the preparation of aryl-keto *α*-amino acids and their corresponding derivatives.

### 2.2. Non-Amino Acid Derivatives as the Reactants

**Scheme 5:**
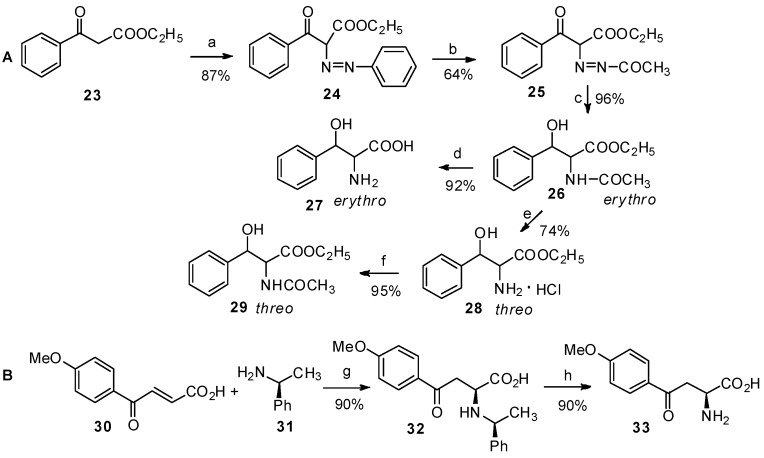
Preparation of aryl-keto *α*-amino acids from non-amino acid derivatives.

It has been reported that the nucleophilic reaction of ethyl benzoylacetate with diazonium is feasible for the preparation of ethyl *α*-phenylazobenzoylacetate [36], which can be used as a useful intermediate for the preparation of *β*-phenylserine (Scheme 5A). And the chiral amine can also be used as a useful amine-donor in Michael addition with benzoylacrylic acid to prepare aryl-keto *α*-amino acid derivatives (Scheme 5B) [10]. 

*Reagents and conditions*: (a) sodium nitrite, HCl, sodium acetate; (b) acetic acid, Zn, acetic anhydride; (c) H_2_, Pd/C, acetic acid; (d) dilute HCl, NH_4_OH; (e) (i) SOCl_2_; (ii) dilute HCl; (iii) HCl/ethanol; (f) sodium acetate, acetic anhydride: (g) methanol, 60 °C; (h) H_2_, Pd/C, ethanol.

Furthermore, the reaction of an organometallic serine derivative and benzaldehyde following further oxidation [37], intramolecular acyl migration reaction of acyclic imides [38], Diels-Alder reaction with amino acid dienophile [39], cross-coupling reactions with aromatic dithianes [40] are also feasible for the preparation of aryl-keto *α*-amino acid derivatives.

The general methods for the preparation of aryl-keto *α*-amino acids based on *α*-amino acid derivatives as the reactants or non-amino acid derivatives are of great importance. Most of these processes are sophisticated, involve cumbersome steps and the use of special catalysts, in addition to requiring time-consuming chiral resolution.

## 3. Preparation of Aryl Keto α-amino Acids by Friedel-Crafts Acylation

Friedel-Crafts acylation is one of the most powerful methods in organic synthesis [41], in which the Lewis acid or Brönsted acid is an indispensible catalyst to promote the reactions [42,43,44,45]. *α*-Amino acids such as aspartic acid and glutamic acid are crucial chiral pool reagents for the preparation of enantiomerically pure compounds. Friedel-Crafts acylation with *α*-amino acid derivatives as acyl donors is a convenient strategy to construct aryl keto *α*-amino acids with the remaining enantiomer configuration. For this purpose, numerous Friedel-Crafts acylations of stoichiometric amounts of *α*-amino acid derivatives were reported with AlCl_3_, HF, or TfOH as catalysts.

### 3.1. AlCl_3_ Catalyzed Friedel-Crafts Acylation

**Scheme 6:**
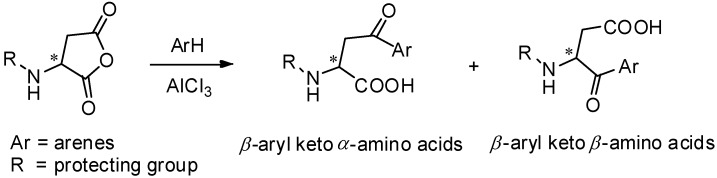
*α*- and *β*- amino acids derived from aspartic anhydrides.

AlCl_3_ is the most commonly used catalyst in Friedel-Crafts acylation [46] and has been widely used for the preparation of aryl keto *α*-amino acids. Aspartic anhydrides, effective and popular acyl donors, are well studied for this purpose. For avoiding the racemization of optically active amino acids during the reaction, amino groups should be protected. Due to the specific structure of anhydrides, the regioselectivity should be taken into consideration. Generally, two kinds of products were obtained as *β*-aryl keto *α*-amino acids and *β*-aryl keto *β*-amino acids (Scheme 6).

**Scheme 7:**
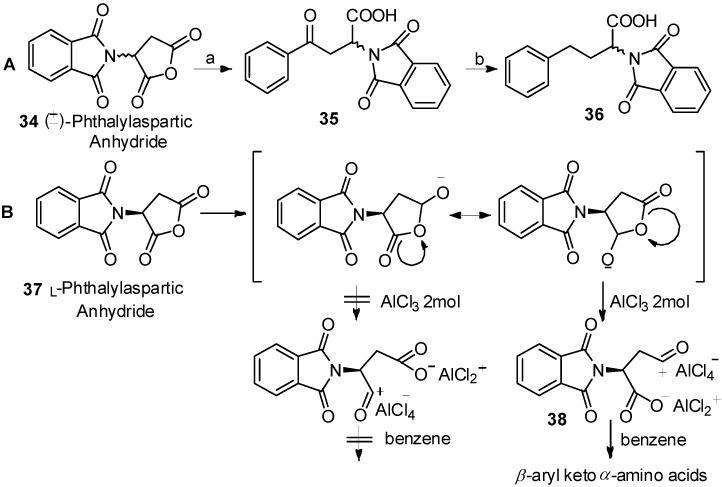
Acylation direction of phthalylaspartic anhydride in Friedel-Crafts acylation.

In 1976, Reifenrath and coworkers reported the Friedel-Crafts acylation of phthalylaspartic anhydride and benzene in the presence of AlCl_3_ (Scheme 7A) [47]. Through comprehensive study, they found that acylation occurred to produce *β*-aryl keto *α*-amino acids **35** as the single products. The same work was reported by Xu, along with the mechanism of the reaction (Scheme 7B) [48]. Due to the strong electron withdrawing effect of the *N*-phthaloylamino group to *α*-carbonyl group, the resonance form for generation of *β*-aryl keto *α*-amino acids **38** was more stable.

*Reagents and conditions*: (a) benzene, AlCl_3_; (b) H_2_/Pt.

**Scheme 8:**
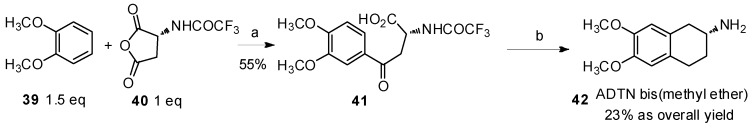
Preparation of ADTN bis(methyl ether) from veratrole by Friedel-Crafts acylation.

Another study was reported by Nordlander and coworkers in 1985 (Scheme 8) [49]. *N*-(Trifluoroacetyl) aspartic anhydride (**40**) was treated with veratrole (**39**) to construct aryl-keto *α*-amino acid derivatives for the preparation of 2-amino-6,7-dihydroxy-l,2,3,4-tetrahydronaphthalene (ADTN) derivative **42**, which was a powerful agonist of dopamine [50].

**Scheme 9:**
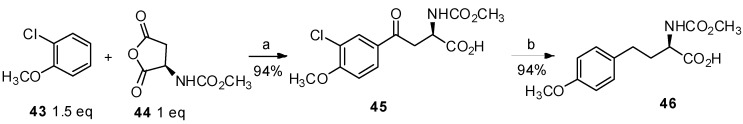
Preparation of homotyrosine derivative from 2-chloroanisole.

As part of developing a strategy for the construction of aryl-keto *α*-amino acids for preparation of dopamine agonists, Melillo and coworkers reported the reaction of 2-chloroanisole with *N*-(methoxycarbonyl) aspartic anhydride (**44**) resulting in the generation of single isomer **45** (Scheme 9) [13]. The key finding in this study was that chlorine as a removable directing group prevented the generation of other isomers to produce para ketone as a single product.

*Reagents and conditions*: (a) AlCl_3_ (2.5 eq), CH_2_Cl_2_; (b) (i) triethylsilane, trifluoroacetic acid; (ii) PCl_5_, CH_2_Cl_2_, SnCl_4_, 0 °C; (iii) triethylsilane, BF_3_·Et_2_O; (iv) K_2_CO_3_, MeOH, H_2_O, reflux.

*Reagents and conditions*: (a) AlCl_3_ (2.5 eq), CH_2_Cl_2_, MeNO_2_; (b) H_2_, Pd/C, *i*-PrOH.

To further study the regioselectivity of anhydrides, Griesbeck and Heckroth published a comprehensive discussion about the reaction between aspartic anhydrides and a variety of aromatics [51]. Several *N*-protected aspartic anhydrides **47a**–**47e**, **37** were reacted with numerous aromatics for investigation of the effect factor for *α*/*β*-selectivity of the products. As a conclusion, both the *N*-protecting groups and aromatics affect the *α*/*β*-selectivity of products (Table 1). 

**Table 1:** Regioselectivity of anhydrides in Friedel-Crafts acylation. 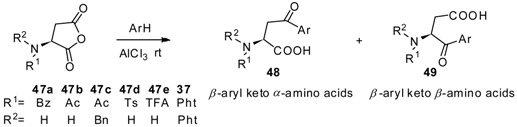

Substrate	arene	Time (h)	α/β-amino acids ratio	Yield (%)	Substrate	arene	Time (h)	α/β-amino acids ratio	Yield (%)
**47a**	benzene	5	55/45	51	**47c**	toluene	5	7/93	47
**47a**	toluene	5	39/61	33	**47c**	o-xylene	15	39/61	52
**47a**	o-xylene	15	30/70	65	**47d**	benzene	5	84/16	76
**47a**	mesitylene	15	100/0	51	**47d**	toluene	5	100/0	71
**47a**	anisole	72	50/50	31	**47d**	o-xylene	15	74/26	83
**47a**	veratrole	96	100/0	18	**47e**	benzene	5	95/5	78
**47a**	naphthalene	15	95/5	44	**47e**	toluene	5	80/20	62
**47b**	benzene	5	95/5	76	**47e**	o-xylene	15	56/44	70
**47b**	toluene	5	64/36	52	**37**	benzene	5	100/0	64
**47b**	o-xylene	15	58/42	67	**37**	toluene	5	88/12	71
**47c**	benzene	5	30/70	55	**37**	o-xylene	15	48/52	83

**Scheme 10:**
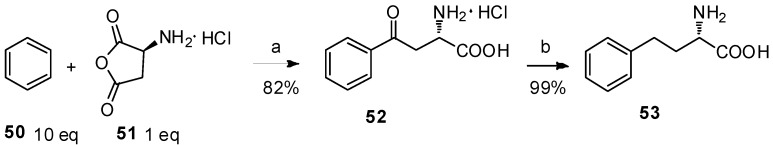
L-Aspartic anhydride hydrochloride as the acyl-donor in Friedel–Crafts acylation.

It is well known that amino-protecting groups of anhydrides not only determine the ratio of *α*/*β*-amino acids but also prevent the racemization of optically active amino acids during the reactions. The most commonly used amino-protecting groups are as follows: ethoxycarbonyl, methoxycarbonyl, trifluoroacetyl, benzoyl, tosyl, acetyl, phthaloyl groups and so on. While the preparation of *N*-protected amino acids is not easy due to the racemization [52], the indispensable deprotection makes large scale-production difficult. In this case, Lin and coworkers reported a precedent of *α*-amino acid anhydride hydrochloride as the acyl-donor in the Friedel–Crafts acylation reaction (Scheme 10) [12]. L-Aspartic anhydride hydrochloride (**51**), prepared from _L_-aspartic acid [53], was treated with anhydrous benzene in the presence of anhydrous AlCl_3_ to form aryl-keto *α*-amino acid hydrochloride **52** in high yield. Upon further hydrogenolysis, L-homophenylalanine (**53**), a versatile building block for the synthesis of pharmaceutical drugs such as angiotensin converting enzyme (ACE) inhibitors, *β*-lactam antibiotics, acetylcholinesterase inhibitors, and neutral endopeptidase inhibitors [54], was obtained in quantitative yield with >99% ee by HPLC.

*Reagents and conditions*: (a) AlCl_3_ (2.6 eq), MeNO_2_, reflux; (b) H_2_, Pd/C, 2 N HCl, 55 °C.

Friedel-Crafts acylation catalyzed by Lewis acids leads to aryl-keto *α*-amino acids as well as their derivatives. However, due to the low solubility of *α*-amino acid derivatives in common organic solvents, reaction yields are low and sometimes a large excess of aromatics, long reaction times, and high temperatures are required. Furthermore, the environmental pollution caused by these catalysts has to be dealt with.

### 3.2. HF Catalyzed Friedel-Crafts Acylation

**Scheme 11:**
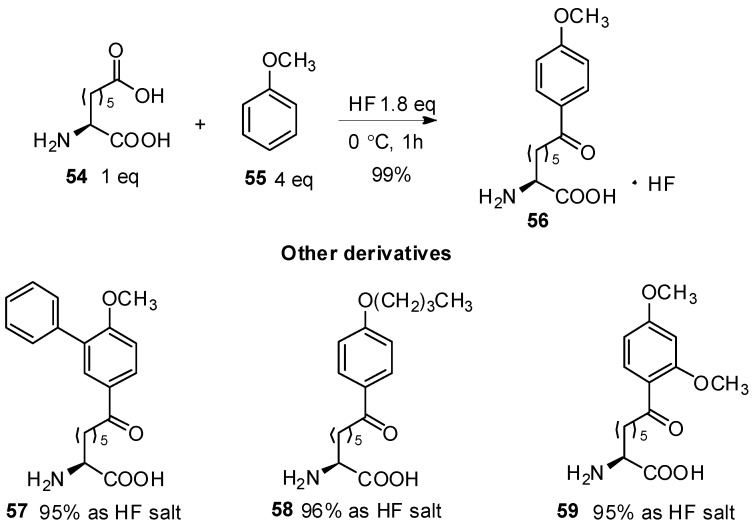
HF as catalyst in Friedel-Crafts acylation.

HF has been used as a catalyst and solvent in Friedel-Crafts acylation for many years [55,56]. It has also been used for the construction of aryl-keto *α*-amino acid derivatives. In 1998, Bednarek reported the preparation of amino acids with aryl-keto functions in the side chains [57]. In this study, *ω*-carboxyl-*α*-amino acid **54** was treated with aromatics such as anisole, 2-methoxybiphenyl, butyl phenyl ether, or 1,3-dimethoxybenzene in the presence of HF as a catalyst. All of the aryl-keto *α*-amino acid derivatives were obtained in high yield in the form of HF salts, which were further incorporated into peptides by conventional methods of coupling (Scheme 11).

### 3.3. TfOH Catalyzed Friedel-Crafts Acylation

TfOH is one of the most important Brönsted acid catalysts used in Friedel-Crafts acylation [44,45,58,59]. Due to its high dissolving capacity, it can easily dissolve most of the *α*-amino acid derivatives. Thus, TfOH catalyzed Friedel-Crafts acylation is a promising strategy to prepare aryl-keto *α*-amino acids from *α*-amino acid derivatives.

In recent years, our research group has been interested in the application of Friedel-Crafts acylation with TfOH as catalyst and solvent. For example, by using TfOH, an effective Friedel-Crafts acylation of *O*- or *C*-arylglucosides was carried out at low temperature for 10 min and no deglycosidation was detected [60]. Introduction of biotin to aromatics through Friedel-Crafts acylation with TfOH afforded numerous biotin derivatives, many of which showed stronger avidin binding potential than biotin [61]. More importantly, synthesis of aryl-keto *α*-amino acids and their derivatives by TfOH catalyzed Friedel-Crafts acylations were well studied.

For convenient synthesis of L-homophenylalanine as well as its derivatives, aryl-keto *α*-amino acid derivatives were prepared as precursors from *N*-TFA-_L_-Asp(Cl)-OMe (**60**) with aromatics, followed by hydrogenolysis and deprotection (Table 2) [62]. *N*-TFA-protected L-Asp anhydride **47e**, one of the most popular acyl donors as described previously, was treated with benzene in the presence of different catalysts such as AlCl_3_, TiCl_4_, H_2_SO_4_, and TfOH. The results indicated that the reaction proceeds well with TfOH in the generation of *α*- and *β*- carboxyl configuration products as a mixture. Further, another acyl-donor, L-aspartic anhydride hydrochloride (**51**), was treated with benzene while no product was detected when TfOH was used as a catalyst. To avoid the formation of *β*-carboxyl configuration products as well as improve the yield from the reaction, *N*-TFA-_L_-Asp(Cl)-OMe [63] was used as an acyl-donor to react with benzene. As expected, 98% of the *α*-carboxyl configuration structure was obtained as a single product within an hour, which indicated that **60** was more reactive than **47e** or **51**. Because **60** contained only one acylation site, the formation of the *β*-carboxyl configuration was prevented. Based on this result, numerous aromatics were reacted with **60** in the presence of TfOH. Through further hydrogenolysis and deprotection, L-homophenylalanine derivatives were obtained with good overall yields.

**Scheme 12:**
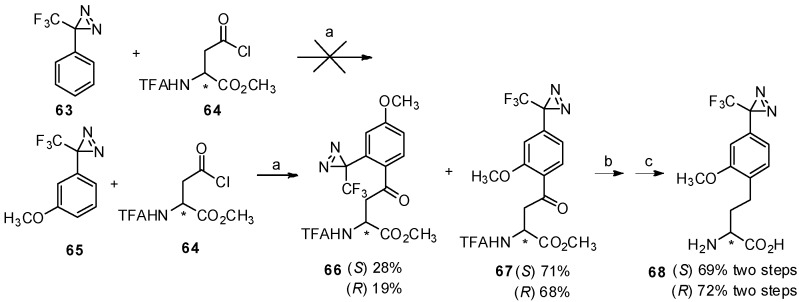
Synthesis of trifluoromethyldiazirinyl homophenylalanine by Friedel-Crafts acylation.

Inspired by this study, an effective synthesis of optically active trifluoromethyldiazirinyl homophenylalanine by Friedel-Crafts acylation with TfOH was reported in 2009 (Scheme 12) [64]. Trifluoromethyldiazirinyl is widely used as a photophore in photoaffinity labeling [65,66]. Upon UV irradiation, the photophore containing ligand can link to receptors or biomolecules by covalent bonds, which makes it feasible for further separation and identification. It has been reported that the diazirine ring is not stable in the presence of Lewis acid over room temperature [67], while catalytic amounts of TfOH do not affect the diazirinyl moiety [68], making it feasible for our work. In this study, *N*-TFA-Asp(Cl)-OMe (**64**) was used as an acyl-donors due to its good reactivity [62]. It was found that 3-phenyl-3-(3-trifluoromethyl)-3*H*-diazirine (**63**) did not react with *N*-TFA-Asp(Cl)-OMe, while higher temperature promoted the decomposition of diazirine [67]. It has been revealed that *m*-methoxy-substituted 3-phenyl-3-(3-trifluoromethyl)-3*H*-diazirine (**65**) not only show higher reactivity, but the methoxy group can be utilized for further introduction of the tag [65]. In this case, 3-(3-methoxyphenyl)-3-(trifluoromethyl)-3*H*-diazirine (**65**) was treated with *N*-TFA-Asp(Cl)-OMe (**64**) in the presence of TfOH at 0 °C and the desired products were obtained in good yield as expected. Due to the decomposition of the *N*-*N* double bond under traditional H_2_-Pd/C conditions [69], the carbonyl group was reduced by the triethylsilane/TFA system and through further deprotection, the desired products were obtained in high yield without racemization. Furthermore, the photolysis properties of these diazirinyl compounds were examined and appropriate values were obtained.

**Table 2:** Friedel–Crafts reaction of anhydrides and stoichiometric amounts of benzene. 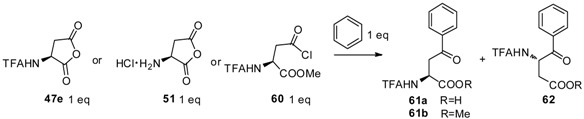

Entry	Donor	Catalyst (eq)	Solvent	Reaction Time (h)	Product Yiled (%)
1	**47e**	AlCl_3_ (8)	CH_2_Cl_2_	1 or 12	0
2	**47e**	TiCl_4_ (90)	neat	1 or 12	1
3	**47e**	H_2_SO_4_ (90)	neat	1 or 12	2
4	**47e**	TfOH (40)	neat	1	**61a** (52), **62** (3)
5	**47e**	Tf_2_OH (25)	neat	1 or 12	0
6	**51**	TfOH (40)	neat	1	0
7	**60**	AlCl_3_ (8)	CH_2_Cl_2_	10	**61b** (50)
8	**60**	TiCl_4_ (90)	neat	1 or 12	0
9	**60**	H_2_SO_4_ (90)	neat	1 or 12	0
10	**60**	TfOH (40)	neat	1	**61b** (98)
11	**60**	Tf_2_OH (25)	neat	1 or 12	0

*Reagents and conditions*: (a) TfOH, 0 °C, 2 h; (b) triethylsilane, TFA; (c) NaOH, MeOH.

**Scheme 13:**
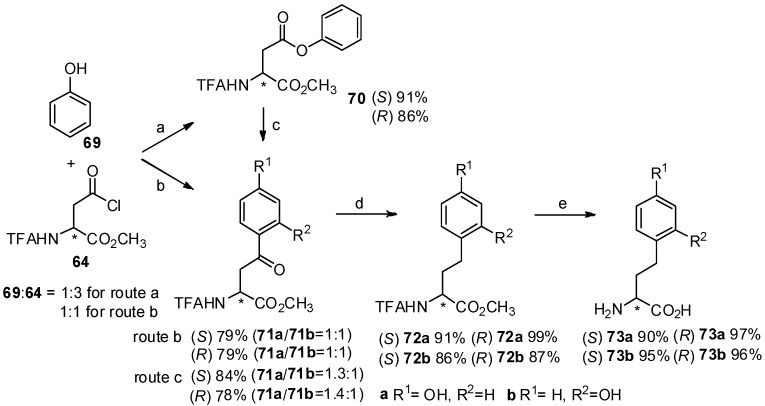
Synthesis of homotyrosines by Friedel-Crafts acylation with TfOH.

Homotyrosine has been found in many biological products [70] and its derivatives play an important role in the synthesis of natural products [71]. For effective synthesis of homotyrosines, our group reported the Friedel-Crafts acylation of *N*-TFA-Asp(Cl)-OMe (**64**) with phenol (**69**) in the presence of TfOH (Scheme 13) [72]. As expected, *p*- and *o*- aroylbenzene derivatives were obtained in high yield within one hour in neat TfOH. Interestingly, *N*-TFA-Asp-OMe *β*-phenyl ester (**70**) as an *O*-acylated product could be obtained from *N*-TFA-Asp(Cl)-OMe with phenol by using diluted TfOH, which can be reconverted to *p*- and *o*-aroylbenzene derivatives upon Fries rearrangement.

*Reagents and conditions*: (a) 3% TfOH-CH_3_CN, rt, 1 h; (b) TfOH, rt, 1 h; (c) TfOH, rt, 16 h; (d) H_2_, Pd/C, rt; (e) 6 N HCl, 80 °C, 6 h.

Further investigation indicated that the reaction time was longer than that of direct Friedel-Crafts acylation with neat TfOH because partial hydrolysis of phenyl ester ocurred, followed by Friedel-Crafts acylation of the carboxylic acid and aromatic hydrocarbon. Finally, two aryl-keto *α*-amino acid derivatives were subjected to further hydrogenolysis and deprotection to form optically pure homotyrosine derivatives.

**Scheme 14:**
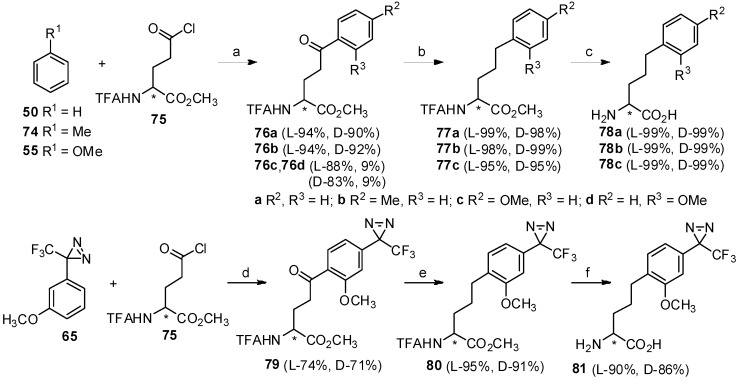
Synthesis of bishomophenylalanine and diazirinyl derivative by Friedel-Crafts acylation.

To further extend the scope of this strategy, glutamic acid was also investigated as the other acyl-donor in the Friedel-Crafts acylation with aromatics (Scheme 14) [14]. Three aromatics (compounds **50**, **74** and **55**) can readily react with *N*-TFA-Glu(Cl)-OMe (**75**) in the presence of TfOH with generation of aryl-keto *α*-amino acids in high yield. Through further reduction and deprotection, corresponding bishomophenylalanines **78a-c** [73] were obtained in high yields without any loss of chiral purity. To study other applications, 3-(3-methoxyphenyl)-3-(3-trifluoromethyl)-3*H*-diazirine (**65**) as the aryl-donor was treated with *N*-TFA-Glu(Cl)-OMe (**75**) in the presence of TfOH. As expected, stereocontrolled aryl-keto *α*-amino acids were obtained, which was consistent with previous reports [64]. Following reduction by the triethylsilane/TFA system and deprotection under alkaline conditions, the diazirinyl moiety-based bishomophenylalanine was obtained in high yield and optical purity. These can be used as important components in photoaffinity labeling [74].

*Reagents and conditions*: (a) TfOH, rt, 1 h; (b) H_2_, Pd/C, AcOH, rt, 1 h; (c) 6 N HCl, 80 °C, 6 h; (d) TfOH, 0 °C, 3 h; (e) Et_3_SiH, TFA, rt, 1 h; (f) NaOH, MeOH, rt, 2 h.

**Scheme 15:**
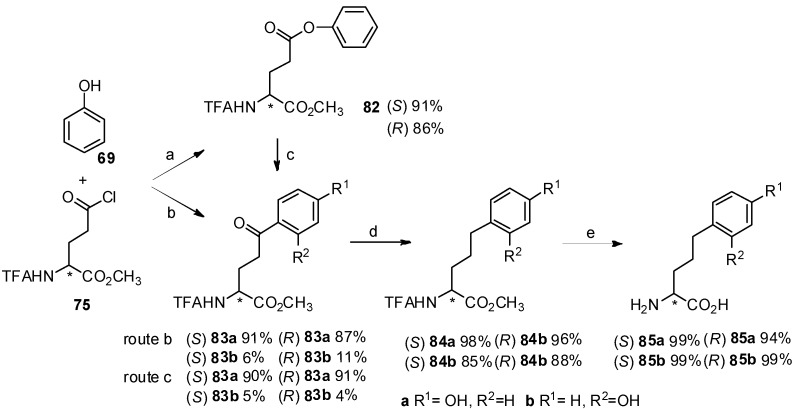
Synthesis of bishomotyrosine by Friedel-Crafts acylation with TfOH.

It is well known that bishomotyrosine is found in the active components of AM-toxin Ⅲ [75] and its convenient synthesis is of great importance. A novel and convenient synthesis of bishomotyrosine through construction of its aryl-keto *α*-amino acid derivative by Friedel-Crafts acylation was reported (Scheme 15) [76]. The corresponding bishomotyrosines were obtained in high optical purity and yield. 

*Reagents and conditions*: (a) 5% TfOH-CH_3_CN, rt, 0.5 h; (b) TfOH, rt, 1 h; (c) TfOH, rt, 2 h; (d) H_2_, Pd/C, rt; (e) 6 N HCl, 80 °C, 6 h

**Scheme 16:**
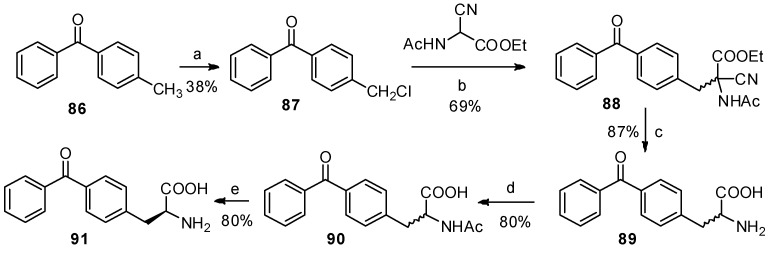
Previous synthesis of *p*-benzoyl-L-phenylalanine from *p*-methylbenzophenone.

Benzophenone is another photophore that is widely used in photoaffinity labeling [77,78]. The benzophenone-based *α*-amino acids are promising structures for protein study. In 1986, Kaure J. C. and co-workers were the first to report the synthesis of *p*-benzoyl-L-phenylalanine (**91**) as a new benzophenone-based amino acid analog (Scheme 16) [79]. Furthermore, *p*-methylbenzoic acid, followed by bromination and treatment with Schiff’s base-activated glycine under basic and phase-transfer conditions, can also be used to prepare *p*-benzoyl-L-phenylalanine derivatives [80]. But there are no reports that benzophenone skeleton was constructed on phenylalanine with Friedel-Crafts benzoylation due to the solubility of phenylalanine in organic solvents.

*Reagents and conditions*: (a) dibenzoyl peroxide, SO_2_Cl_2_; (b) K_2_CO_3_, acetone; (c) (i) 8 N HCl; (ii) hot water, 1 N NaOH, (d) acetic anhydride, NaOH; (e) (i) NH_4_OH, aspergillus acylase I, toluene; (ii) 5 N HCl, 70 °C, Celite; (iii) 1 N NaOH.

**Scheme 17:**
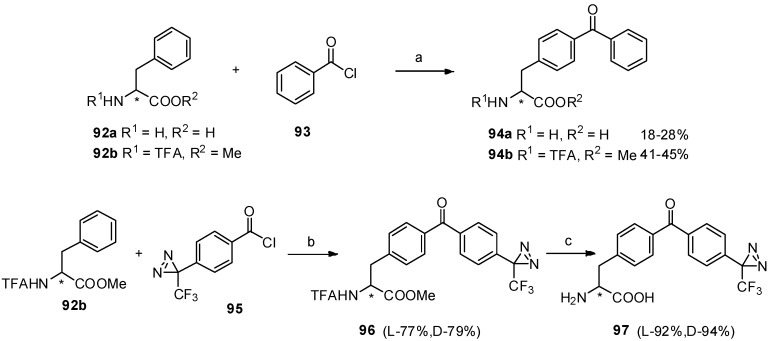
Synthesis of benzophenone based *α*-amino acid derivatives and two photophores based structures by Friedel-Crafts acylation with TfOH.

For convenient synthesis of benzophenone-based *α*-amino acid derivatives, we carried out Friedel-Crafts acylation between phenylalanine derivatives **92a-b** and diazirinylbenzoyl chloride **95** in the presence of TfOH [81]. Furthermore, two photophore (benzophenone and diazirine) based structures (**97**) were prepared as an interesting study and their detailed analysis of photo-irradiation were also investigated, which may contribute to the investigation of peptide-receptor interactions in photoaffinity labeling (Scheme 17).

*Reagents and conditions*: (a) TfOH; (b) TfOH, 0 °C; (c) NaOH.

**Scheme 18:**
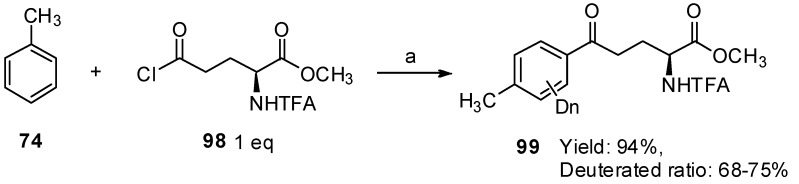
Friedel–Crafts reaction and hydrogen/deuterium exchange with TfOD.

TfOH is not only an excellent solvent but also an effective catalyst in the Friedel-Crafts acylation of *α*-amino acid derivatives and aromatics. Inspired by these results, we investigated the utilization of TfOD for this study, to ensure the status of the generated acylation (Scheme 18) [82]. *N*-TFA-_L_-Glu(Cl)-OMe (**98**) was treated with toluene in the presence of TfOD at room temperature for 1 h, and aryl-keto *α*-amino acid derivatives were obtained in high yield. Interestingly, it was found that hydrogen/deuterium exchange occurred on the aromatic ring and the deuterium incorporation was calculated to be 68%–75% based on ^1^H-NMR spectroscopic analysis.

*Reagents and conditions*: (a) TfOD, rt, 1 h.

In comparison with TfOH, TfOD performs the same role in Friedel-Crafts acylation between *α*-amino acid derivatives and aromatics. On the other hand, TfOD is also the deuterium-donating reagent, which effectively promotes hydrogen/deuterium exchange on aromatic *α*-amino acids. As a part of developing a novel strategy, a series of hydrogen/deuterium exchanges for aromatic *α*-amino acids and their corresponding peptides were also performed in the presence of TfOD. Furthermore, TfOD-catalyzed hydrogen/deuterium exchange was also carried out on some cross-linkable *α*-amino acid derivatives, which will contribute to effective analysis of biological functions of bioactive peptides and proteins by MS [83].

## 4. Conclusions

In conclusion, aryl-keto *α*-amino acid derivatives, excellent building blocks in organic chemistry and biochemistry, can be readily prepared under different conditions using several strategies. Among them, Friedel-Crafts acylation in the presence of AlCl_3_, HF, or TfOH(D) is one of the most convenient and effective methods. As a strong Brönsted acid, TfOH behaves not only as a catalyst, but also as an excellent solvent for dissolving *α*-amino acid derivatives that are not easy to dissolve in common organic solvents. Following further hydrogenolysis and deprotection, a lot of useful non-proteinogenic amino acids and functional structures such as photophores containing *α*-amino acid derivatives are obtained with high optical purity and yield.
